# Friedreich ataxia in Norway – an epidemiological, molecular and clinical study

**DOI:** 10.1186/s13023-015-0328-4

**Published:** 2015-09-04

**Authors:** Iselin Marie Wedding, Mette Kroken, Sandra Pilar Henriksen, Kaja Kristine Selmer, Torunn Fiskerstrand, Per Morten Knappskog, Tone Berge, Chantal ME Tallaksen

**Affiliations:** Department of Neurology, Oslo University Hospital, Ullevaal, 0407 Oslo Norway; Department of Medical Genetics, Oslo University Hospital, Ullevaal, 0407 Oslo Norway; University of Oslo, Faculty of Medicine, Oslo, Norway; Department of Clinical Science, University of Bergen, Bergen, Norway; Center for Medical Genetics and Molecular Medicine, Haukeland University Hospital, Bergen, Norway

**Keywords:** Friedreich ataxia, FRDA, Frataxin, Ataxia, Autosomal recessive, Norway

## Abstract

**Background:**

Friedreich ataxia is an autosomal recessive hereditary spinocerebellar disorder, characterized by progressive limb and gait ataxia due to proprioceptive loss, often complicated by cardiomyopathy, diabetes and skeletal deformities. Friedreich ataxia is the most common hereditary ataxia, with a reported prevalence of 1:20 000 – 1:50 000 in Central Europe. Previous reports from south Norway have found a prevalence varying from 1:100 000 – 1:1 350 000; no studies are previously done in the rest of the country.

**Methods:**

In this cross-sectional study, Friedreich ataxia patients were identified through colleagues in neurological, pediatric and genetic departments, hospital archives searches, patients’ associations, and National Centre for Rare Disorders. All included patients, carriers and controls were investigated clinically and molecularly with genotype characterization including size determination of GAA repeat expansions and frataxin measurements. 1376 healthy blood donors were tested for GAA repeat expansion for carrier frequency analysis.

**Results:**

Twenty-nine Friedreich ataxia patients were identified in Norway, of which 23 were ethnic Norwegian, corresponding to a prevalence of 1:176 000 and 1:191 000, respectively. The highest prevalence was seen in the north. Carrier frequency of 1:196 (95 % CI = [1:752–1:112]) was found. Homozygous GAA repeat expansions in the *FXN* gene were found in 27/29, while two patients were compound heterozygous with c.467 T < C, L157P and the deletion (g.120032_122808del) including exon 5a. Two additional patients were heterozygous for GAA repeat expansions only. Significant differences in the level of frataxin were found between the included patients (N = 27), carriers (N = 37) and controls (N = 27).

**Conclusions:**

In this first thorough study of a complete national cohort of Friedreich ataxia patients, and first nation-wide study of Friedreich ataxia in Norway, the prevalence of Friedreich ataxia in Norway is lower than in Central Europe, but higher than in the last Norwegian report, and as expected from migration studies. A south–north prevalence gradient is present. Based on Hardy Weinberg’s equilibrium, the carrier frequency of 1:196 is consistent with the observed prevalence. All genotypes, and typical and atypical phenotypes were present in the Norwegian population. The patients were phenotypically similar to European cohorts. Frataxin was useful in the diagnostic work-up of heterozygous symptomatic cases.

**Electronic supplementary material:**

The online version of this article (doi:10.1186/s13023-015-0328-4) contains supplementary material, which is available to authorized users.

## Background

Friedreich ataxia (FRDA) is an autosomal recessive spinocerebellar disorder, and is reported to be the most frequent of the large and heterogeneous group of hereditary ataxias in Europe, the Middle East, the Indian subcontinent in South Asia, and North Africa [1]. Degeneration of the dorsal root ganglia and spinal roots, dorsal columns, spinocerebellar tracts, corticospinal tracts and cerebellar dentate nucleus give rise to the clinical picture of progressive limb and gait ataxia, proprioceptive loss, absent tendon reflexes and dysarthria [2–5]. The disease is often further complicated by extra-neurological manifestations such as cardiomyopathy, diabetes and skeletal deformities [6]. FRDA is reported worldwide with the exception of sub-Saharan Africa and southeast Asia [7], and the prevalence in Central Europe is estimated to be between 1:20 000 to 1:50 000. Its presence in Norway remains, however, poorly documented. Whereas Skre reported a prevalence of 1:100 000 in the western part Norway in 1975 [8], Koht did not find any ethnic Norwegian cases in southeast Norway in 2007 [9], and Erichsen et al. found a prevalence of 0.15:100 000 in the same region in 2008 [10]. However, molecular testing was not available until 1996 [11], thus Skre’s report may have included phenocopies, for instance patients with ataxia due to *POLG* mutations [12]. Nonetheless, a FRDA prevalence of 1:208 000 is expected in Norway based on migration studies, which is higher than in other Nordic countries [13] (Table 1). Health authorities depend on reliable epidemiological data for planning and making strategies for present and future health care, especially for implementation and funding of future treatments. Though there is still no specific curative treatment available for FRDA today, several drugs are under current investigation. It is of paramount importance as for all rare diseases to identify patients – both to ensure the best present clinical practice follow-up, and to be able to offer them state of the art new treatments when these emerge. A thorough characterization of the cohort is also required for participation in future clinical trials.

**Table 1:** Published and estimated FRDA prevalence numbers from Nordic countries

Country	Prevalence	Predicted FRDA prevalence^a^ [13]
Norway [8, 10]	1:100 000–1:1 350 000	1:208 860
Denmark [63]	1:140 000	1:132 540:
Sweden [42]	1:420 000	1:410 027
Finland [43]	1:750 000	-
Iceland [64]	1:93 600	-

^a^ (based on R1b haplotype)

The classic FRDA phenotype, as described by Harding’s clinical criteria, included onset before the age of 25, progressive ataxia, absent knee and ankle jerks, axonal neuropathy and dysarthria [6]. The phenotypical spectrum of FRDA widened after the discovery of the disease-causing mutations in the *FXN* gene. Today, 15 % of cases are described with onset later than 25 (Late-onset FRDA (LOFA)) or 40 years of age (very late-onset FRDA (VLOFA)), and other atypical presentations as FRDA with retained reflexes (FARR) are also reported [14]. 96-98 % of FRDA cases are caused by homozygous GAA triplet repeat expansions in the first intron of the *FXN*-gene, while the remaining present compound heterozygosity with GAA-expansions and point mutations or deletions [11, 15–17]. The mutations lead to decreased transcription of frataxin mRNA, subsequently leading to lower levels of the mitochondrial protein frataxin. The biological role of frataxin is not yet completely understood [18], but it is known to have a role in iron-sulphur (Fe-S) cluster biogenesis in the mitochondria. Lack of frataxin causes iron accumulation and impaired electron transport in the respiratory chain in the mitochondria, further leading to increased formation of toxic free radicals and oxidative stress [19]. The frataxin level in multiple cell types, including blood cells and fibroblasts, appears to be correlated with the frataxin level in disease-affected tissues [20]. This level is low in FRDA patients, while healthy carriers show intermediate frataxin levels [21]. It is suggested that frataxin level may be useful in diagnostics or follow up of patients [22], as it represents a standardized biochemical marker.

Disease causing alleles of *FXN* usually have between 70–1500 GAA repeats [4, 23]. Fifty % of the variation in phenotype severity is estimated to be due to the GAA expansion size [24], with the size of the smaller allele showing the strongest correlation [3, 25]. Frataxin levels are also found to be inversely correlated with GAA repeat length [21]. Normal alleles can be divided into Short Normal (SN) alleles containing up to 12 GAA repeats in the majority of healthy individuals, and Long Normal (LN) alleles with up to 33 GAA repeats. Alleles between 34 and 65 GAA repeats can act as unstable premutation alleles. Both LN alleles, premutation alleles and expanded alleles have shown linkage disequilibrium for the same nearby alleles, suggesting a common origin [7, 26]. LN alleles reaching the instability threshold estimation of 34 may therefore function as a mutation reservoir in the population giving rise to new pathogenic expansions in intergenerational transmission [27].

In order to ensure the best clinical practice and follow-up, the epidemiological, clinical, and molecular characteristics of FRDA patients in Norway was investigated. The first objective of this study was to establish the prevalence and carrier frequency of FRDA in Norway through a population-based epidemiological study. The second objective was to establish a Norwegian control material for frataxin measurements in whole blood, and assess the level in whole blood in all of affected subjects, carriers and healthy controls. The last objective of this study was to assess GAA repeat expansion sizes, both in the patients and in the background population. In addition, we wanted to relate the patients’ genotypes to their clinical and molecular phenotypes, and to compare the Norwegian patients with other case series.

## Methods

### Patients and controls

**Fig. 1:**
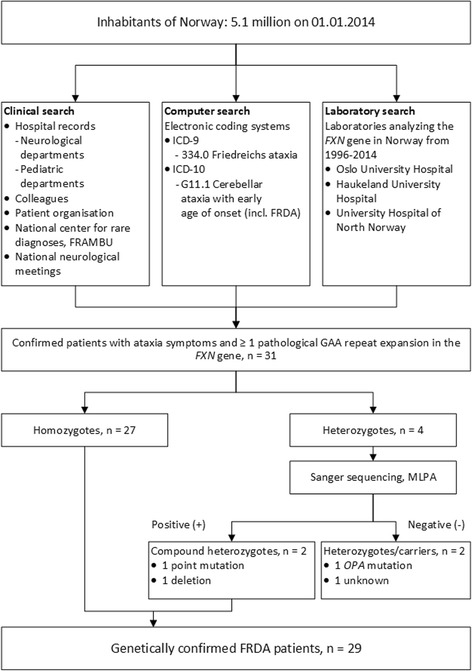
Flowchart showing patient ascertainment and the Friedreich ataxia population in Norway

As shown in Fig. 1, three main sources were used to identify FRDA patients:

- Clinical search: At the time of the study there were altogether 22 different public hospitals in Norway which had either a general pediatric (20 departments) or a general neurological (19 departments) department, or both. Six of these were university hospitals. All departments were contacted by postal mail first in September 2011, with follow-up by telephone during 2011. Patients’ organizations and the National center for rare diagnoses, so called Frambu, were contacted during the second half of 2011, and the project was presented at the national neurological meeting in 2013 to ensure good recruitment of patients. The investigators were continuously informed about newly diagnosed patients from these sources until prevalence date, November 30th, 2014.
- Computer search: Searches were performed based on electronic coding systems in hospital archives, with searches for “334.0 Friedreich ataxia” in ICD-9, and G11.1 “Cerebellar ataxia with early age of onset (incl. FRDA)” in ICD-10. Searches were performed during the second half of 2011.
- Laboratory search: The three laboratories offering *FXN* – gene analysis in Norway at prevalence date, November 30th, 2014, were contacted twice; first in October 2011, then at prevalence date to identify newly diagnosed patients within the study period. All individuals with positive findings from the establishment of the method to this date were further investigated.

Eligible for inclusion were all affected subjects with ataxia symptoms, at least one pathological expansion > 66 GAA-repeats in the *FXN* gene, and alive on November 30, 2014 (n = 31). Patients with no GAA repeat expansions in the *FXN* gene were excluded. Healthy carriers consisted of 1^st^ degree relatives of the patients aged > 18 years who were heterozygous for mutations in the *FXN* gene (n = 37). The controls (n = 27) were healthy age-matched individuals, recruited among friends and colleagues. All subjects were interviewed and examined by the neurologist IMW.

1376 healthy anonymous ethnic Norwegian blood donors from Oslo were used in the carrier frequency study.

The data of the Norwegian background population are from Statistics Norway [28] on January 1st, 2014.

### Ethics, consent and permissions

Written informed consent was obtained from all study participants or their authorized guardians. The study was approved by The Regional Committee for Research Ethics (Ethical agreement n°129/04011), in Norway.

### Clinical examination

All identified patients who met the inclusion criteria were invited to participate in the study. Clinical data were systematically registered according to the SPATAX network’s diagnostic form for spinocerebellar degeneration [29, 30] including the Scale for the Assessment and Rating of Ataxia (SARA) [31]. Associated symptoms like signs of cardiomyopathy at echocardiogram, scoliosis, diabetes, dysphagia, hearing loss and psychiatric problems were carefully registered by interview and the patients’ medical charts, as shown in Table 2.

**Table 2:** Clinical characteristics of included Friedreich ataxia patients in Norway at time of study

Family – patient	Gender	Age at onset (in years)	Duration (in years)	SARA^a^	Presenting symptom	Disability stage^b^	Extensor plantar response	Reduced virbratory sense	Dysphagia	Dysarthria	Scoliosis	Diabetes	Depression	Heart involvement	Frataxin pg/mcg	GAA repeats, Allele 1	GAA repeats, Allele 2
1-1	F	6	11	19	Cardiomyopathy	4	Bilateral	y	n	y	y	n	y	y	0.126	700	700
2-1	M	8	8	19	Unsteady	4	Bilateral	y	y	y	y	n	n	n	0.150	570	870
3-1	F	7	10	23	Unsteady	5	Bilateral	y	y	y	y	n	n	n	0.135	740	1000
4-1	M	18	11	25.5	Unsteady	5	Unilateral	y	y	y	y	n	y	y	0.305	640	640
4-2	M	2	15	31	Unsteady	6	Equivocal	y	y	y	y	n	n	y	0.045	700	700
5-1	M	4	17	28	Unsteady	6	Bilateral	y	n	y	y	y	n	n	0.079	700	700
6-1	F	6	8	14.5	Unsteady	4	Unilateral	y	n	y	y	n	y	n	0.132	700	700
7-1	M	3	20	33	Unsteady	6	Bilateral	y	y	y	y	n	y	y	0.042	800	800
8-1	F	6	6	11.5	Unsteady	3	Normal	n	n	n	y	n	n	n	0.084	800	800
9-1	M	17	9	11	Unsteady	4	Equivocal	y	n	n	n	n	n	n	0.207	370	700
10-1	M	6	12	12.5	Unsteady.	3	Bilateral	y	y	y	y	n	y	y	0.130	770	770
11-1	M	15	4	9	Unsteady	2	Normal	n	y	y	y	n	n	n	0.307	340	940
12-1	M	10	59	31	Unsteady	6	Unilateral	y	y	y	y	n	y	y	0.256	340	800
13-1	F	12	19	32	Unsteady	6	Bilateral	y	y	y	y	n	n	n	0.020	700	1040
13-2	F	10	23	38.5	Unsteady	6	Bilateral	y	y	y	y	n	y	n	0.068	740	740
14-1	F	20	11	10.5	Unsteady	3	Bilateral	y	y	y	y	n	n	n	0.904	400	400
14-2	F	20	9	13.5	Unsteady	3	Bilateral	y	y	y	y	n	y	n	0.826	340	340
15-1	M	15	7	28	Unsteady	6	Bilateral	y	y	y	n	n	n	y	0.103	670	870
16-1	M	5	9	12.5	Unsteady	3	Normal	y	y	y	y	n	n	y	0.179	470	870
17-1	F	5	6	11	Unsteady	2	Normal	y	n	n	n	n	n	n	0.058	700	700
18-1	M	15	33	33	Unsteady	6	Bilateral	y	y	y	y	n	n	n	0.180	540	940
19-1	F	17	17	24	Unsteady	5	Unilateral	y	y	y	n	n	n	n	0.323	340	800
20-1	F	5	6	6.5	Unsteady	2	Bilateral	y	n	y	y	n	n	y	0.109	700	700
21-1	M	13	18	31	Unsteady	5	Bilateral	y	y	y	y	n	n	y	0.215	700	700
22-1	M	4	21	32	Unsteady	6	Bilateral	y	y	y	y	y	y	y	0.031	740	c.467T>C
23-1	F	7	33	30	Unsteady	6	Bilateral	y	y	y	y	n	n	y	0.126	770	g.120032_ 122808del
24-1	F	3	9	14	Cardio myopathy	3	Normal	y	y	y	y	n	n	y	na	na	na
Summary mean (SD) or %	M: 52 % F: 48 %	9.6 (5.7)	15.2 (11.6)	21.6 (9.6)	Unsteady: 93 % Cardiomyopathy: 7%	4.4 (1.5)	Bilateral: 59 % unilateral: 15 % Equivocal: 7 % normal: 19 %	93 %	93 %	89 %	85 %	7 %	33 %	48 %	0.198 (0.214)	615 (161.8)	759 (160.6)

^a^SARA (Scale for the Assessment and Rating of Ataxia)

^b^Disability stage 1-7: 1:(no disability), 1:(no functional handicap but signs at examination, 2:(mild, able to run, walking unlimited), 3:(moderate, unable to run, limited walking without aid), 4:(severe, walking with one stick), 5:(walking with two sticks), 6:(unable to walk, requiring wheelchair), 7:(confined to bed)

### Molecular analyses

All patients were tested for GAA triplet repeat expansions to confirm their genetic diagnosis. The methods used were standard polymerase chain reaction (PCR), for detection of alleles in normal range, and Southern blot or TP PCR (Triplet Repeat Primed PCR) for detection of expanded alleles. All patients that were heterozygous for the GAA triplet repeat expansion, were subsequently analyzed by Sanger sequencing of the coding region including exon-intron boundaries, for detecting of sequence variants, and by Multiplex ligand-dependent probe amplification (MLPA) for detection of deletions/duplications. The reference sequence NM_000144.4 was used.

Analyses for expanded GAA repeats were performed by standard PCR method, using AmpliTaq Gold 360 (Applied Biosystems, Foster City, USA). Primer sequences and PCR conditions used for sequencing are available on request. TP PCR was performed as described by Ciotti P.et al. [32]. Sanger sequencing was performed using BigDye Terminator v.3.1 Cycle Sequencing Kit (Applied Biosystems, Foster City, USA). SALSA MLPA P316 (MRC Holland, The Netherlands) was used according to the manufacturer’s instructions to detect single or multiple deletions/duplications of the *FXN* gene. Size determination of expanded alleles in patients and carriers was analyzed by Southern blot [33].

#### GAA expansion size

Determination of GAA repeat lengths of the shortest allele (GAA1) and longest allele (GAA2) was done in all patients, carriers and controls for phenotypic characterization. The number of GAA repeats in the normal range was determined by PCR amplification followed by analysis by capillary electrophoresis. Expanded alleles were analyzed by Southern blot. The GAA repeat sizes as measured by PCR were calculated relative to a standard size ladder (GS500-ROX35-250, Applied Biosystems). In Southern blot analyses, smear length was converted to GAA repeat size by dividing kb with three.

#### Transmission instability

The degree of meiotic instability of GAA repeats was assessed by analyzing differences in GAA repeat expansion sizes in parents and children. As information of the parental origin of alleles was not present, the minimal alteration possible was presumed in every transmission, as previously done [34]. Only expansions or retractions above a threshold of 0.3 kb (100 GAA repeats) were registered, because most of the larger expansions appeared as smears on the blot, and it was difficult to estimate the exact number of repeats. The results are shown in Additional file 1.

#### Carrier frequency study

DNA from 1376 healthy anonymous blood donors was analyzed by TP PCR, and subsequently standard PCR for those with expanded alleles to exclude homozygosity. The normal alleles were sorted into Short Normal (SN – fewer than 12 GAA-repeats) and Long Normal (LN – 12 to 34 GAA-repeats), and premutation alleles (35–66 GAA-repeats) to find the proportion of possible unstable LN alleles.

### Frataxin protein level

Frataxin level in whole blood was measured by Frataxin protein quantity dipstick assay kits (MitoSciences (MSF31), Abcam, Cambridge, UK). Whole blood was collected from patients, carriers and controls in K2 EDTA BD Vacutainer tubes (REF 454209). The patients’ and carriers’ samples were stored in room temperature for 2 to 7 days while they were sent to our center by postal mail, immediately frozen at −20 °C at reception, and thereafter stored at −80 °C. The time of storage of the control samples were matched to the patient/carrier samples. Recombinant human frataxin full-length protein (ab110353, Abcam) was diluted 1:2 for generation of standard curve. Briefly, 50 μl of whole blood was mixed with 150 μl of ice cold extraction buffer. After clarifying the extracts by centrifugation at 14500 RPM for 20 min at 4 °C, the supernatant was collected and 0.5 μl of Sigma P8340 protease inhibitor (Sigma, St. Louis, MO) was added. The samples were analyzed as described in the protocol provided by the manufacturer [21, 35]. The immunecaptured frataxin was quantified with a Hamamatsu immunochromato reader (MS1000 Dipstick reader, Abcam). Samples were analyzed in triplicates, and the median value was selected for the final calculation. A logarithmic standard curve ranging from 0.4 – 100 ng/ml frataxin was used. The total protein quantity in each lysate was determined using the BCA Protein Assay Kit (23225, Pierce Biotechnology, Rockford, IL).

### Statistical analysis

Statistical analysis of clinical data and the frataxin assay was performed using Statistical Package for the Social Sciences (SPSS) v.22 (IBM, Armonk, NY, USA) or Graph Pad Prism (Graph Pad Software, Inc., San Diego, CA). A p-value of < 0.05 was set as the threshold for significance. Chi square or Fisher’s exact test were used when comparing the epidemiological proportions (north vs. south, ethnic vs. non-ethnic Norwegians and male vs. female transmission). Fisher’s exact test was used when expected frequencies were less than five in one or more cells. Mann Whitney U test was used when comparing continuous variables like clinical characteristics between groups of patients (ethnic vs non-ethnic Norwegians, male vs female), as well as in comparisons of frataxin level between patients, carriers and controls after Kruskal-Wallis test was performed. Correlations between disease duration, age at onset, neurological severity (SARA score), molecular characteristics (GAA repeat length, frataxin level) and the presence of non-neurological complications like scoliosis, heart involvement and diabetes were assessed with Spearman’s rho. In correspondence with Cohen, the size of correlation coefficient r is interpreted as 0.10-0.29 = small, 0.30-0.49 = medium, and >0.50 = large. To examine the relationship between frataxin level and the significant predictors we used multiple regression analysis. Receiver operating characteristic (ROC)-curves were used to select cutoff values for the frataxin measurements, calculating area under the ROC curve, sensitivity and specificity.

Power analysis calculating the size of the blood donor sample for carrier frequency analysis was done with the help of a statistician.

## Results

### Epidemiological data

#### Prevalence

As shown in Fig. 1, 31 patients from 28 unrelated families with ataxia symptoms and at least one allele with pathological GAA repeat expansion in the *FXN*-gene were identified as alive on prevalence date. In 30/39 of the contacted hospital departments, either the head of department or the doctor in charge of ataxias consulted with their colleagues whether they were aware of FRDA patients, and answered our query. In eight of the remaining departments, electronic searches based on ICD diagnosis coding in hospital archives including both inpatients and outpatients, were performed. Only one smaller department in the south of Norway did not respond. However, this department only has an out patients clinic for neurology. Moreover neurologists have been referring their ataxia patients to our university hospital. In all six university hospitals in Norway, representatives from all neurological and pediatric departments answered the query. Additionally, electronic searches were performed in four departments in university hospitals. Due to legal reasons these searches were done by local clinicians. The electronic searches in Oslo University Hospital (OUS) were made by IMW. 17 of all 31 patients from all over the country were identified in the OUS’ archives, truly mostly due to the tertiary specialist function of OUS for these rare ataxia patients. Through ICD-9, 14 patients were identified at OUS, only two of them eligible for inclusion and alive. The G11.1-code in ICD-10 showed 121 hits at OUS; 17 of the 18 patients eligible for inclusion were alive, including the two identified by ICD-9 codes.

The clinical departments, the National center for rare diagnoses and patients’ organizations informed continuously about all new diagnosed patients between 2011 and November 30th 2014.

Among the different sources, 30/31(97 %) patients were identified through colleagues in clinical departments, 21/31 (68 %) were identified through the National center for rare diagnoses, Frambu, and 27/31 (87 %) were registered in genetic departments. Thus clinical departments were the most powerful source, but only two patients were identified exclusively through this source. All other patients were identified through at least two different sources. Twenty-seven were confirmed homozygous for GAA repeat expansions. Four patients were heterozygous for GAA repeat expansion. Two of these patients were identified as being compound heterozygous; one had a mutation c.467 T < C, p.L157P (Table 2: Patient 22–1) and the other had a deletion (Patient 23–1), both in *trans*. The deletion was confirmed by long-range PCR to be consistent with the deletion of 2776 bp (g.120032_122808del) including exon 5a. The remaining two heterozygous patients had normal findings by Sanger sequencing and MLPA of the *FXN*-gene, and were not included in the prevalence number. One of these also carried a previously reported pathogenic *OPA1* mutation [36]. Thus, FRDA was genetically confirmed in 29 of 31 individuals (15 males, 14 females), while two individuals were heterozygous for GAA repeat expansion.

**Fig. 2:**
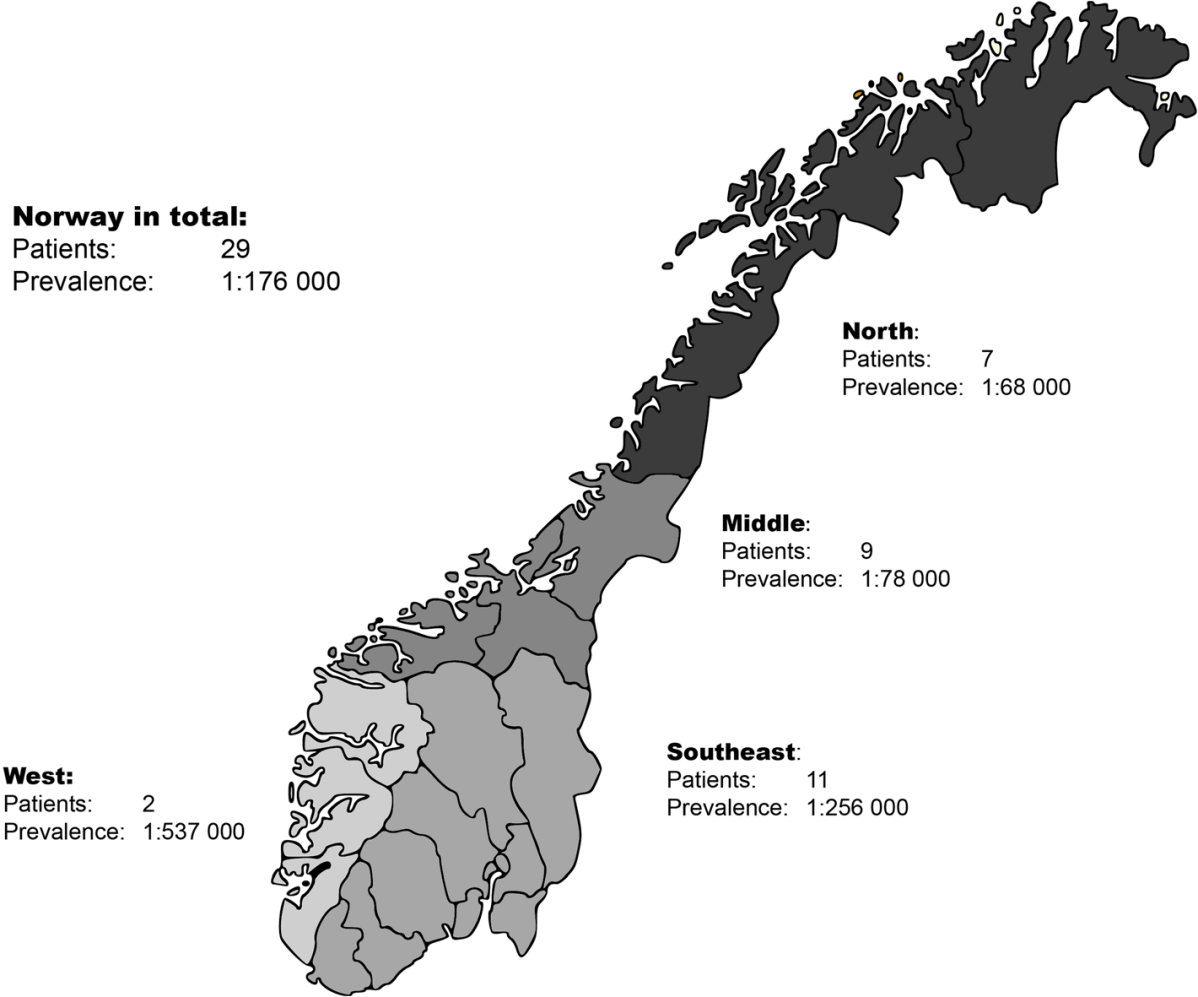
Total and regional FRDA prevalence in Norway on 30.11.2014

Of the confirmed FRDA patients 23 were ethnic Norwegians, three were Caucasians from other European countries while three were non-Caucasians from the Middle East and North-Africa. As shown in Fig. 2, total prevalence in Norway was 1:176 000 (population: 5 109 056 on 1.1.2014), with a significant difference between north and south (southeast + west) Norway (*p* = 0.0008). Prevalence was similar in the ethnic Norwegian population (1:191 000 (population: 4 398 591 on 1.1.2014)), and non-ethnic Norwegian population (1:118 410 (population: 710 465 on 1.1.2014)) (*p* = 0.29).

#### Carrier frequency study

Seven expanded FXN alleles were found among 2752 alleles from healthy ethnic Norwegian blood donors in Oslo, corresponding to an observed carrier frequency of 1:196 (95 % CI = [1:752–1:112]). The observed FRDA prevalence among ethnic Norwegians in southeast Norway was 1:314 714 (population on 1.1.2014: 2 203 000). Hardy Weinberg equilibrium (HWE) estimation of the carrier frequency in the southeast Norwegian population is 1:281, which is within the confidence interval of the observed carrier frequency. In the entire ethnic Norwegian population the HWE estimated carrier frequency is 1:210.

There were 454 long normal alleles (LN) with an expansion size of 12–34 repeats, of which 38 were longer than 27 repeats (Table 3). Three (0.11 %) alleles were between 34 and 66 repeats, classified as premutation alleles.

**Table 3:** Distribution of the frequencies of the GAA repeat sizes in normal alleles in 6 different populations

Normal alleles		Norwegian (present study)	Finnish [43]		Cuban [65]	Indian [66]	Caucasian [67]	French [26]
			North	South				
GAA repeat sizes	5-12	83.5 %	83 %	79.5 %	89.1 %	94.1 %	80 %	83 %
	13-34	16.5 %	17 %	20.5 %	10.9 %	5.9 %	20 %	17 %
Alleles range		6-34	6-27	7-27	5-31	7-16	5-23	7-34

### Characterization of phenotype

Twenty-seven of 29 genetically confirmed FRDA patients (25 homozygous and 2 compound heterozygous) agreed to participate in the clinical part of the study. These had a median age on prevalence day of 22 years, and a median age at onset of 7 years, with no late onset cases (>25 years of age). Seven patients had a positive family history of FRDA (siblings) and 3 patients reported consanguinity (Table 4).

**Table 4:** Reports of phenotypes in Friedreich ataxia patients in Norwegian patients compared to other case series

Clinical feature	Harding [6]	Filla et al. [37]	Dürr et al. [3]	Schöls et al. [34]	Lamont et al. [14]	2Delatycki et al. [38]	McCabe et al. [39]	Salehi et al. [40]	Reetz et al. [41]	Present study
Country of study	UK	Italy	France	Germany	UK	Australia	Ireland	Iran	Europe (EFACTS)	Norway
Year of publication	1981	1990	1996	1997	1997	1999	2000	2014	2015	2015
No of patients	115	80	140	38	56	51	58	22	592	27
Genetically confirmed diagnosis	No	No	Yes	Yes	Yes	Yes	Yes	Yes	Yes	Yes
Age at onset (years), Mean(range),*Median[interquartile range]* ^*a*^	10.52 (1.7-27)	11.6 (2-23)	15.5 (2-51)	14.15 (5-36)	3 to 30	10.5 (SD±6.4) (1-26)	-	10.8 (2-23)	15.7 (SD±10.4), *13 [9-19]*	9.6 (2-20)
Age at examination (years), Mean(range),*Median[interquartile range]* ^*a*^	-	25 (8-55)	-	-	-	-	-	-	33.9 (SD±10.2) *32 [23-43]*	24.8 (11-69)
Disease duration (years), Mean(range),*Median[interquartile range]* ^*a*^	22.0 (SD±12.8) (2-61)	13.4	15.5	19.7 (SD±8.8) (5-42)	13.6 (SD±9.9)	-	-	-	18.3 (SD±10.4) *17 [10-25]*	15.2 (4-59)
Male:female	1:1.2	1:0.7	1:1.1	1:1.4	-	1:0.8	-	1:0.6	1:1.2	1:0.9
SARA (Standardized Rating Scale of Ataxia), mean(range), *Median[interquartile range]*	-	-	-	-	-	-	-	-	*23 [13-21]*	21.6 (6.5-38.5), *23 [13.8-32.3]*
Disability stage (1-7)^b^, Median	-	-	-	-	-	-	-	-	5	5
GAA repeats shortest allele, Mean(range), *Median[interquartile range]* ^*a*^	-	-	630 (SD±230)	800 (66-1360)	2-5 kb	739 (SD±191)	762 (333-1053)	594 (247-981)	*648 [384-800]*	614.6 (340-800), *700 [556-844]*
GAA repeats longest allele, Mean(range), *Median[interquartile range]* ^*a*^	-	-	890 (SD±230)	-	2-5 kb	973 (SD±162)	885 (534-1200)	-	*912 [789-1050]*	759.2 (340-1040), *755 [670-840]*
Frataxin level in whole blood (pg/μg)	-	-	*-*	-	-	-	-	-	*-*	0.198
Homozygous GAA-repeat expansion (%)	-	-	-	-	-	-	-	100	97	93
Family history of FRDA (%)	-	23.4	-	-	-	-	-	-	32	20
Consanguinity (%)	-	28.1	-	-	-	-	-	-	-	7
Intake of Idebenone (%)	-	-	-	-	-	-	-	-	27	52
Gait ataxia (%)	100	100	100	100	100	100	100	100	-	100
Limb ataxia (%)	99	94	99	100	100	100	-	100	-	100
Dysarthria (%)	97	84	91	100	91	95	93	95	-	89
Lower limb areflexia (%)	99	100	87	84	87	98	86	100	-	93
Extensor plantar reflexes (%)	89	75	79	95	96	73.5	93	90	-	74
Reduced vibratory sense (%)	73	91	78	83	87	88	89	63	-	93
Scoliosis (%)	79	94	60	84	-	78	84	-	-	85
Foot deformity (%)	55	90	55	82	-	74	79	54	-	81
Gaze-evoked nystagmus (%)	20	29	40	39	-	-	40	45	-	18.5
Fixation instability (%)	-	-	-	69	-	-	-	-	-	51.8
Saccadic pursuit eye movements (%)	12	-	30	32	-	-	52	-	-	67
Dysphagia (%)	-	30	27	76	-	-	-	-	-	78
Diabetes (%)	10	14	32^c^	6	-	8	7	4.5	-	7
Cardiomyopathy (echocardiography) (%)	66^d^	28	63	75	-	65	67	-	-	48 (n=26)
Hearing loss (%)^e^	8	9	13	39	-	-	-	-	-	26
Reduced vision (%)^e^	18		13	6	-	-	-	9	-	22
Psychiatric symptoms (%)^f^	-	-	-	-	-	-	-	-	-	37
Atypical (%)	-	-	24	25	-	8	-	-	-	7
Wheelchair bound (%)	72	43	-	78	-	55	-	45	-	52
Age when wheelchair-bound (years)	25.1 (SD±15.5)	26.3 (SD±7.8)	26.3	24.0 (SD±5.7)	-	19.0 (SD±6.4)	-	-	-	20.9 (SD±7.7)
Disease duration to wheelchair-bound (years)	15.5 (SD±7.41)	13.8 (SD±5.8)	10.8 (SD±6)	11.3 (SD±4.1)	-	10.1 (SD±4.4)	-	-	-	11.1 (SD±6.4)
Prognosis index^g^	0.31	0.31	-	0.25	-	-	-	-	-	0.29

^a^Unless otherwise stated (SD=Standard deviation)

^b^Disability stage 1-7: 1:(no disability), 1:(no functional handicap but signs at examination, 2:(mild, able to run, walking unlimited), 3:(moderate, unable to run, limited walking without aid), 4:(severe, walking with one stick), 5:(walking with two sticks), 6:(unable to walk, requiring wheelchair), 7:(confined to bed)

^c^impaired glucose tolerance?

^d^in Hardings study based on abnormal ECG, except for 9.6 % where ECG changes regarded insignificant

^e^Based on information given by the patients

^f^Requiring psychiatric treatment

^g^Prognosis index: mean disease duration in all patients in the case series/proportion of wheelchair-bound patients in the case series

Twenty-five of 27 patients had typical FRDA according to Harding’s diagnostic criteria [6], while two were atypical due to brisk reflexes consistent with FARR (Table 2: Patient 14–1 and 14–2). The mean SARA score was 21.6. Regarding non-neurological complications, two patients had confirmed diabetes; one of them was compound heterozygous with a point mutation. 23/27 had scoliosis; five of them had been treated with spine surgery. Cardiomyopathy was confirmed in 14/26 patients by echocardiography, mostly as asymptomatic hypertrophy. However, four had atrial fibrillation, further complicated by stroke in one patient in his fifties (Patient 12–1), and cardiac infarction in one patient at age 25 (Patient 22–1). Nine patients had depression necessitating treatment. All clinical parameters as reported in Table 4 were similar in ethnic and non-ethnic Norwegians. There were no significant gender differences in disease duration, SARA score, age at onset, cardiomyopathy, scoliosis, depression, frataxin level nor GAA repeat lengths. There were no obvious differences in the Norwegian patient series compared to the previously described case series reported in Table 4 [3, 6, 14, 34, 37–41].

### Molecular and biochemical characterization

Molecular analyses were done in 29 patients (including two heterozygous for GAA repeat expansion), 37 healthy 1^st^ degree carrier relatives (19 mothers, 13 fathers, 3 siblings, and 2 children of the patients) and 27 healthy controls (mean age 25.9 years, male–female ratio: 1:2.7).

#### GAA repeat expansion size

In 29 patients (27 GAA repeat expansion homozygous and two compound heterozygous) the GAA1 repeat length ranged from 340 to 740, GAA2 (n = 25) from 340 to 1040, while healthy carriers showed 240 to 1040 repeats in the expanded allele (Additional file 1). Alleles from 27 healthy controls ranged from 7 to 22 GAA repeats.

#### Frataxin protein level

**Fig. 3:**
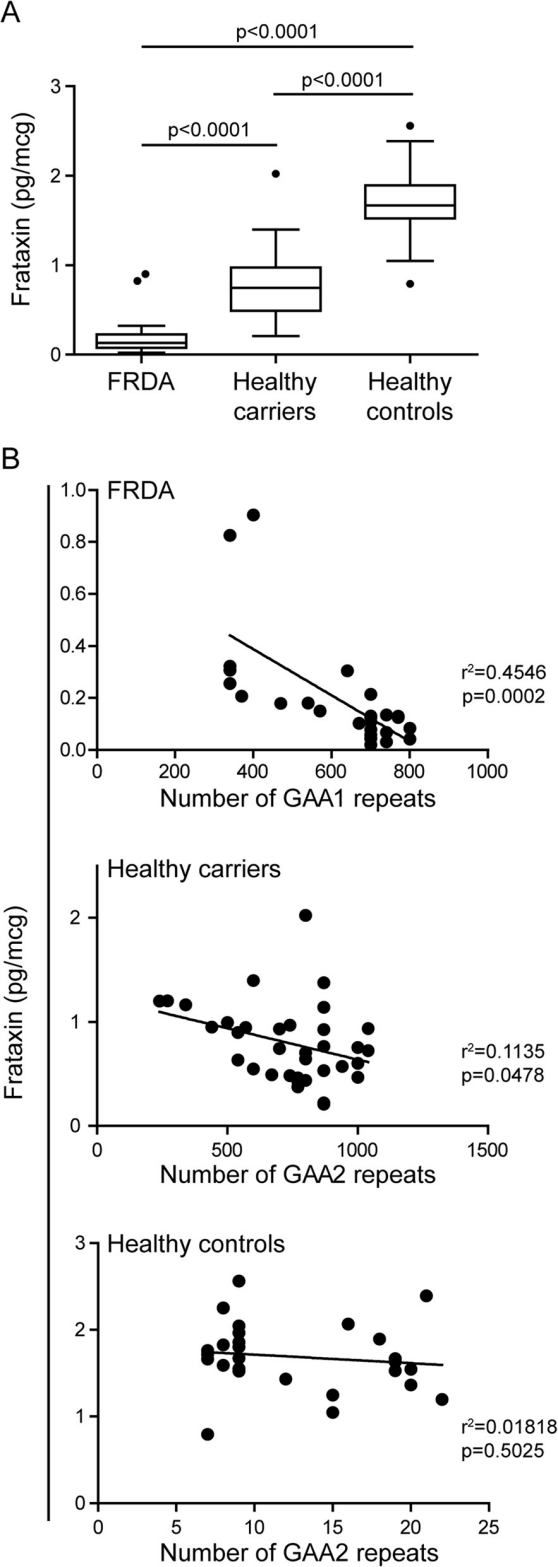
Frataxin levels in whole blood in FRDA patients, carriers and controls. The frataxin level is significantly different between the three groups and correlates with number of GAA1 repeats in FRDA patients and healthy carriers. The levels of frataxin were measured in blood from FRDA patients (N = 26), healthy carriers (N = 37) and healthy controls (N = 27). **a** The Tukey box plot shows pg frataxin per μg (mcg) total protein. The whiskers extends to a maximum of 1.5 x interquartile range (IQR) beyond the edge of the box (“Tukey’s inner fence”). Values outside these fences represent outliers and are marked as dots. **b** Linear regression analyses were performed to analyze the correlation between frataxin levels (pg/mcg) in blood and number of GAA1 repeats in FRDA patients (upper plot), number of GAA2 repeats in healthy carriers (middle plot) and number of GAA2 repeats in healthy controls. R^2^ = coefficient of determination

**Fig. 4:**
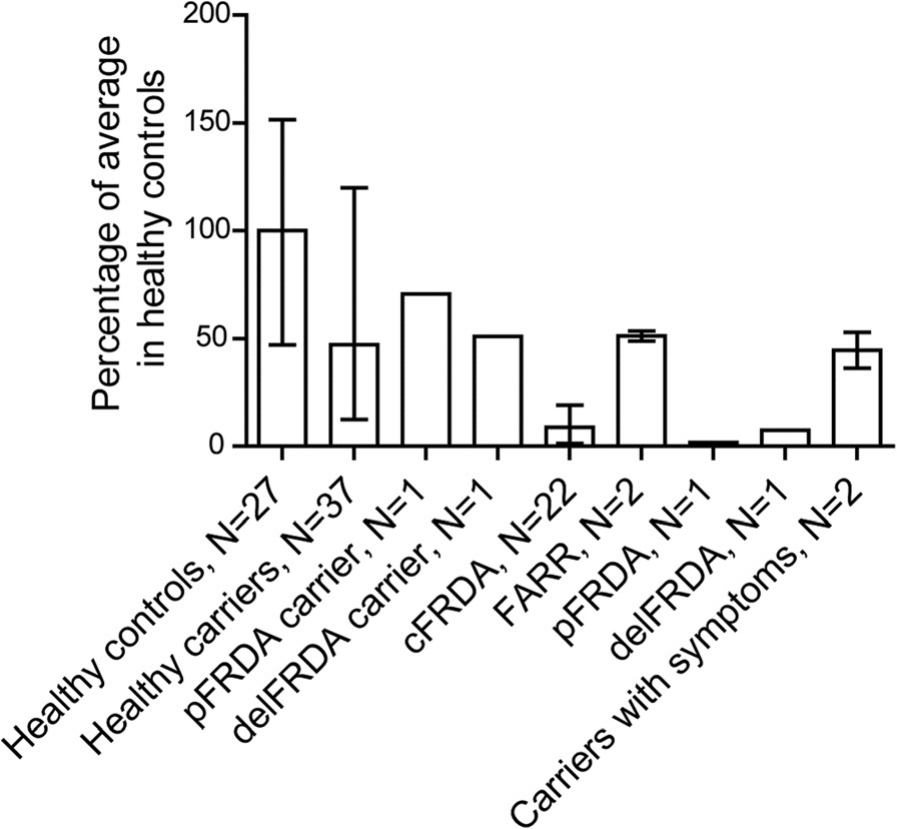
Frataxin amounts in percentage of average amounts in healthy controls in subgroups of carriers and patients. The graph displays the mean relative frataxin amounts for the indicated subgroups of carriers and patients; Healthy controls (N = 27), Healthy carriers (N = 37), pFRDA carrier = healthy carrier with heterozygous point mutation in the *FXN* gene (N = 1), delFRDA carrier = healthy carrier with heterozygous deletion in the *FXN* gene (N = 1), cFRDA = classic FRDA (N = 22), FARR = Friedreichs ataxia with Retained Reflexes (N = 2), pFRDA = compound heterozygous with point mutation (N = 1), delFRDA = compound heterozygous with deletion (N = 1), Carriers with symptoms (N = 2)

Figures 3a and 4 show that frataxin levels in whole blood differed significantly between FRDA patients, *FXN* mutation carriers and healthy controls. Two outliers in the patient group were FARR patients with a mean frataxin level of 0.86 pg/μg, while the classic homozygous GAA repeat expansion patients (cFRDA) had a mean frataxin level of 0.14 pg/μg. Healthy carriers showed a mean frataxin level of 0.8 pg/μg, and healthy controls showed a level of 1.69 pg/μg.

ROC curves were constructed to select cut-off values for frataxin levels in order to assess the ability of the measurements to separate between “disease” and “no disease” (Additional file 2). Two curves were constructed, first to distinguish between FRDA patients from controls and carriers combined, and secondly to distinguish between FRDA and carriers combined from healthy controls, as previously shown [22]. Comparison of FRDA patients versus controls/carriers, resulted in a 92.3 % sensitivity and a 96.9 % specificity for a cut-off value of frataxin of 0.35 pg/μg (*p* < 0.0001, area = 0.968), while comparison of FRDA patients/carriers with controls, resulted in a 92.1 % sensitivity and a 92.6 % specificity for a cut-off value of 1.19 pg/μg (*p* < 0.0001, area = 0.968).

#### Correlations

Disease duration was strongly positively correlated to SARA (r = 0.817, *p* = <0.0001) and clinical stage (r = .784, *p* = <0.0001).

At the molecular level, frataxin level was strongly positively correlated to age at onset (r = 0.745, *p* = <0.0001) and age at wheelchair bound (r = 0.856, *p* < 0.0001). Frataxin was also negatively correlated to neurological severity with total SARA score (r = −0.390, p = 0.049), as well as to the sub-scores SARA nose-finger test (r = −0.440, *p* = 0.025), SARA stance (r = −0.404, *p* = 0.041), and SARA sitting (r = −0.439, *p* = 0.025). Additionally, frataxin was inversely correlated to the presence of impaired touch and prick sense (r = −0.544, *p* = 0.004).

A strong inverse correlation was found between GAA1 length and age at onset (r = −0.634, *p* = 0.001). No correlation was found between SARA score and the length of any of the expanded alleles. Frataxin level was also strongly negatively correlated to the GAA1 length (r = −0.674, *p* = 0.0002) (Fig. 3b), but not to GAA2 length (data not shown) in FRDA patients. In healthy carriers a moderate and significant negative correlation between frataxin level and allele size was present(r = −0.337, *p* = 0.048), but not in normal controls (r = −0.135, *p* = 0.503) (Fig. 3b).

The only factor that was related to extra-neurological features was the moderate negative association between heart involvement and age at onset (Spearmans rho −0.396, *p* = 0.041). Otherwise, no relationships were found between extra-neurological features (foot deformities, scoliosis or heart affection) and age at onset, disease duration, clinical severity, GAA sizes, or frataxin level.

A multiple linear regression analysis was performed to identify possible predictors of frataxin level. Due to the strong association between GAA1 and age at onset, two multivariable models were needed to avoid multicollinearity problems. In model 1 which included age at onset and SARA as possible predictors, 61 % of the variance within frataxin level was explained by these two factors (R^2^ = 0.608), with age at onset (regression coefficient: 0.026 (95 % CI = [0.016–0.037]), *p* = <0.001) as the strongest predictor, while SARA showed lower influence (regression coefficient: −0.07 (95 % CI = [−0.013– -0.001]), *p* = 0.035). Model 2 included two variables, GAA1 size and SARA, and these factors explained 48 % of the variance within frataxin level (R^2^ = 0.484). Within this model, GAA1 size was the strongest predictor (regression coefficient −0.001(95 % CI = [−0.001– -0.0004]), *p* = 0.01), while the effect of SARA was not significant (regression coefficient −0.004 (95 % CI = [−0.011–0.004]), *p* = 0.331) in the model.

#### Transmission instability

The degree of meiotic instability of GAA repeats in the transmission from parents to children was assessed in 21 families with transmission analysis from healthy carrier parents to affected children in 11 mother/father/child-trios and 9 mother/child duos. In addition, the transmission from an affected parent to healthy carrier children was analyzed in 2 father/child duos (Additional file 1). The maximum expansion size was 200 and the maximum retraction size was 300 GAA repeats. As haplotype phasing was not available, the minimum possible change was registered, which probably represents an underestimate [34]. No cases of expansion from premutation alleles to expanded alleles were observed.

## Discussion

In this first study of Friedreich ataxia in the entire Norwegian population, the prevalence result interestingly supports prevalence estimates based on migration studies, although an unexpected south–north prevalence gradient is seen. The carrier frequency estimate is in concordance with the estimated prevalence, suggesting a good diagnostic coverage. This study is not only the first comprehensive study of genetically confirmed FRDA in Scandinavia, but also one of the first clinical and molecular descriptions of a complete national collection of FRDA patients.

### Epidemiology

The study confirms the presence of FRDA in ethnic Norwegians, contrary to what was suggested previously [9]. The thorough epidemiological approach described in Fig. 1 covered all Norwegian regions, including the corresponding university hospitals. Thus, all identified Norwegian FRDA patients who were symptomatic and diagnosed at the time of the study was probably identified. The Norwegian health care system is almost exclusively public, well organized and all cases of suspected FRDA are referred to a few specialized hospital departments and laboratories. Moreover, the study was done in parallel with recruitment of all spinocerebellar ataxias in southeast Norway. Some cases may however have been missed, either as undiagnosed or presymptomatic cases at the time of the study, or due to misdiagnosed patients lost to follow up. Also, some late onset and atypical cases may have been undiagnosed or missed. However, the observed carrier frequency in southeast Norway of 1:196 was within the range of what is expected from the prevalence in the corresponding population in this region, supportive of a true low prevalence as found.

In our study the most sensitive case-finding source was through all clinical departments in Norway, through which 97 % of all cases where identified. Due to Norway’s small size, it was feasible to contact all clinical departments. However, as there was no perfect overlap to other sources of patient identification, a multi-source approach for identifying patients proved valuable, and is recommended in future studies. Surprisingly, the genetic departments had only registered 87 % of all patients, but four patients were diagnosed abroad. The largest collection of patients found by one single source was through the National center for rare diagnoses, Frambu, where almost 70 % of the patients were registered. Frambu is a nationwide competence center for rare disorders, offering information, support and courses for FRDA families and the results suggest that it is widely used by the patients.

According to recent studies, the GAA expansion mutation may have originated in Africa up to 24 000 years ago [7], and later gone through a genetic bottleneck in Southern France during the last ice age. This is supported by the observation that FRDA prevalence in Europe is decreasing along the migration route of this population, characterized by the R1b haplotype proportion [13]. The theory implicates a decrease in prevalence in a north-eastern direction, consistent with the decreasingly lower prevalence rates in Sweden and Finland, with 21 reported patients in Sweden [42], and even less in Finland [43]. The prevalence of 1:191 000 in ethnic Norwegians is surprisingly close to the estimate of 1:208 000 from these migration studies [9].

The higher prevalence in the north of Norway is, however, unexpected according to this theory, especially as the R1b haplotype proportion is lower in the north [44]. None of the non-ethnic Norwegian patients lived in the north, making the south–north gradient even more pronounced among ethnic Norwegians. Nevertheless, the same pattern is observed in Finland and has been explained by a northern migration in the 15^th^ century [43]. In Norway, the mutation may have been brought northwards during one of several periods of high migration from south to north. In addition, the population has historically been less mobile in the north of Norway due to geographical circumstances, and the level of consanguinity among ethnic Norwegians seems higher here compared to the south of Norway [45–48]. This could favor preservation of recessive mutations in the population, thus contributing to this regional difference. However, the numbers are small, and coincidences may carry a statistically large impact, leading to a type I error.

Surprisingly, 16.5 % of the alleles from almost 1400 healthy blood donors were longer than 13 repeats, i.e. on the same level as France, where FRDA is much more prevalent [26]. This level is seen in several Caucasian populations, also in low-prevalent Finland. The Finnish sample, however, contained no alleles larger than 27 repeats, and the missing reservoir of expansion-prone premutation alleles was suggested as a possible reason for the very low Finnish FRDA prevalence [43]. One cannot exclude that the higher FRDA prevalence in Norway as compared to Finland may be partly due to the larger proportion of premutation alleles, thus maintaining a certain level of carriers. Carriers of premutation alleles are in general estimated to be far less common than carriers of pathogenic expanded alleles, as seen also in the blood donor population with 0.11 % permutation carriers vs 0.25 % mutation carriers. Thus, premutation allele expansion in FRDA transmission is very unusual [1]. In our patient population we have registered 33 of 56 possible transmissions, and in these no expansions from permutation alleles were found. However, expansions from premutation alleles may have occurred in the carrier population, in which most of the pathogenic alleles in the population occur.

### Clinical characterization

Norwegian patients showed similar ataxia severity as the recently published large European EFACTS cohort using the FRDA validated SARA scale [41, 49] for comparison. When compared to other case series, our patients show closest resemblance to the German phenotypes, in particular regarding frequencies of skeletal deformities and dysphagia. However, except for Acadian patients, who for unknown reasons show a milder clinical picture [25], no noticeable differences have previously been found across ethnicities in FRDA patients. This is also observed within Norway, where ethnic and non-ethnic Norwegians were clinically similar.

Almost 40 % of the patients reported depression that necessitated treatment, in line with the recent report by daSilva, where 30 % of FRDA patients fulfilled the diagnostic criteria for major depression [50]. Depression and psychiatric complications are increasingly being recognized in FRDA, and the high number in this study clearly demonstrates the need for a systematic assessment and awareness of depression in future studies and clinical practice.

In general, the Norwegian health care system provides patients with good physiotherapeutic and medical follow-up, and interestingly, the proportion of Idebenone users was higher than in the EFACTS cohort [41]. Although Idebenone has not shown convincing therapeutic effect in larger studies [51, 52], the availability of other supportive treatments could lead to the expectation of a relatively good prognosis in the Norwegian patients. With lack of longitudinal data, prognosis may be estimated by the relationship between disease duration and severity of symptoms, or the cardiomyopathy, which is known to be the most common cause of premature death in FRDA patients [53]. Age at onset to wheelchair-bound and proportion of cardiomyopathy were, however, disappointingly similar to other case series and no sign of prognostic differences could be found. Social support and economic resources in addition to physical impairment have been shown to have an impact in health related quality of life in FRDA patients [54]. Thus, it is possible that quality of life evaluation could show differences compared to patients in other countries, however, this was not included in the protocol.

As atypical cases are previously described to represent up to ¼ of FRDA patients [3, 14, 34], the present proportion of atypical patients was surprisingly low with only two patients (7 %). These were two siblings with hyperreflexia, disease onset at the age of 20, and a milder course, consistent with the clinical entity of FARR that usually shows a better prognosis than classic FRDA [55]. The small number of atypical patients may be coincidental due to our small sample size, but it is also possible that atypical presentations are more often missed and may have stayed undiagnosed.

### GAA repeats

The previously described and inverse relationship between GAA1 length and age of onset [3, 56], as well as to frataxin level [21], was confirmed. Hyper-expanded disease-associated repeats are known to show instability in transmission [27, 56, 57], and may explain some of the large phenotypic interfamilial variability in neurological severity and age at onset, as seen in family 4 in Table 2.

### Frataxin

Whole blood from patients contained on average 1/10 of the amount of frataxin found in healthy controls, with a large variance, ranging from 1–50 % of control level. As frataxin measurements are done in research settings only, large differences are seen in the absolute frataxin levels reported from different laboratories and in different tissues. In agreement with Sacca *et al.*, both absolute values and relative values compared to healthy controls are reported [58]. From our experience different product batches gave different absolute frataxin concentrations despite the use of recombinant frataxin as standard curve reference, and a widespread use of the method in clinical practice will require establishment of local reference values or lower inter-laboratory variance. Frataxin is proposed to be a standardized biochemical disease marker that may have advantages over GAA repeat size, by overcoming genotypic allele size related difficulties like somatic variability/mosaicism, and compound heterozygosity, as well as being suitable for monitoring treatment. Even though a correlation is previously found between frataxin level in affected and unaffected tissues [20], measurements of frataxin in readily available non-affected tissues, such as blood and cheek swabs, will only represent a surrogate marker of the level in target tissues such as neurons, cardiomyocytes and pancreatic cells. In addition, the dipstick assay measures the frataxin 81–210 isoform, but several isoforms of frataxin exist in different cells [59], with different biochemical and functional properties. Thus, it has been suggested that an expression of isoform ratios between patients and healthy individuals may be an even better surrogate outcome marker in the future [60]. Despite all these limitations, a clear correlation between frataxin levels in whole blood and clinical ataxia severity, as well as age at onset and GAA1 repeat size, could be confirmed, with age at onset being the strongest predictor of frataxin level. No correlations were found between frataxin and non-neurological symptoms, which could be due to tissue-specific isoform differences in the cardiac and pancreatic cells [59]. Interestingly, a negative correlation between GAA1 length and frataxin level was not only found in the patients, but also in healthy carriers. In normal individuals, previous reports about an association between GAA repeat length and frataxin level have been conflicting [58, 61]. In this series of 27 healthy controls there is a trend towards a correlation between GAA repeat length and frataxin level. Other still unknown factors are probably contributing to the large frataxin level variance in the healthy state.

Although frataxin levels are significantly different at group level for patients and carriers, it is interesting to note that the two clearly outlier cases with higher frataxin level in the patients’ group belonged to the two atypical FARR cases, which is consistent with previous reports [22]. The use of frataxin measurement in the diagnostic work-up of heterozygote individuals has previously been suggested by Sacca et al. [58, 62], and was also found useful in this study. Based on ROC curve analyses (Additional file 2), criteria of the measured frataxin were classified and contributed to the diagnostic work-up of four patients who all showed an ataxia phenotype, but who were heterozygous for the GAA repeat expansion. One patient had a known missense mutation in *trans*, c.467 T < C, L156P, previously described in Sweden [15]. Consistent with its location in the C-terminus of the mature frataxin it resulted in a typical phenotype, and the frataxin level was well below the patient frataxin cut-off value of 0.31 pg/μg. The second patient heterozygous for a GAA repeat expansion had frataxin levels in the lower range of levels seen in the patient group (7 % of average controls), and subsequent MLPA resulted in detection of the deletion g.120032_122808del, including exon 5a. The deletion is previously described in Germany [16], with a very similar phenotype with age of onset at 9, hypertrophic cardiomyopathy, foot deformity, scoliosis and axonal sensory neuropathy. Even though this patient showed a typical FRDA phenotype, the definite genetic FRDA diagnosis was first made after MLPA, preceded by a frataxin level in the lower patients’ range.

The two other heterozygous patients showed frataxin levels within the carrier range. One had an *OPA1* mutation, causing mitochondrial dysfunction. This patient had a complex phenotype, with sensory ataxia and axonal neuropathy compatible with FRDA, but in addition a severe optic atrophy, making the clinical picture more consistent with a severe *OPA1* phenotype [36]. Even though optic atrophy does occur in FRDA patients it is uncommon, and a primary OPA1 diagnosis is further supported by frataxin level only in the carrier range. The other patient showed signs of childhood onset dorsal column dysfunction consistent with FRDA, but had additional atypical features like early onset cerebellar atrophy, intellectual deterioration, hyperreflexia and epilepsy as well as elevated blood lactate, clinically consistent with mitochondrial dysfunction. Based on their frataxin levels these two patients may be coincidental carriers of the GAA triplet repeat expansion, as is expected to be seen in 1:200 Norwegians. However, based on the knowledge of a mitochondrial role of frataxin it could be tempting to hypothesize a possible contribution of the relative frataxin deficiency in heterozygotes, to the severity of their mitochondrial disease.

### Future perspectives

In the literature, FRDA is now clinically and genetically well described, and in recent years the understanding of the molecular pathogenesis has improved. This has paved the way for possible treatments, and several clinical trials on different compounds are on-going. Frataxin levels are increasingly being used as an outcome measure in clinical trials, and it is important to establish reference values and baseline data in well characterized patients, as we aimed to do for the Norwegian population. A thorough characterization of the nation’s patients is crucial for participation in clinical trials, as well as for implementing treatments when eventually available. This study could pave the way for inclusion of the highly motivated Norwegian patients in future clinical trials.

## Conclusions

This study is not only the first comprehensive study of genetically confirmed Friedreich ataxia in Scandinavia, but also one of the first studies that describes in detail both clinical and molecular features of a complete national collection of FRDA patients. The strength of this study is the inclusion of all diagnosed patients within a country, which reduces selection bias and thereby gives a good overview of the spectrum of the disease. FRDA is present in Norway with a prevalence of 1:176 000 (1:191 000 among ethnic Norwegians), as expected from clinical reports and genetic population studies. An unexpected significant south–north gradient is seen. The assessment of frataxin proved useful for diagnostics, especially in heterozygous symptomatic cases.

## Additional files

Additional file 1:
**Meiotic instability of GAA repeats analyzed by differences in GAA repeat expansion sizes in parents and children.** (DOCX 22 kb) Additional file 2:
**ROC curves based on measuring frataxin from whole blood in FRDA patients, carriers and healthy controls.** (DOCX 38 kb)

## Abbreviations

FRDA: Friedreich ataxia
GAA1: shortest GAA repeat expansion
GAA2: longest GAA repeat expansion
MLPA: Multiplex ligand-dependent probe amplification
SARA: Scale for the Assessment and Rating of Ataxia SARA
