# Supplementary material for: Friedreich ataxia in Norway – an epidemiological, molecular and clinical study
Source: Orphanet J Rare Dis. 2015 Sep 4;10:108. doi: 10.1186/s13023-015-0328-4 (PMC4559212; doi:10.1186/s13023-015-0328-4)
Supplement: Additional file 1: — Meiotic instability of GAA repeats analyzed by differences in GAA repeat expansion sizes in parents and children. (DOCX 22 kb) [file 13023_2015_328_MOESM1_ESM.docx]

Legend: Only expansions or retractions ≥ 0.3 kb (100 GAA repeats) were registered, because most of the expansions appeared as smears on the blot, and it is difficult to estimate the exact number of repeats. In total, 10 expansions, 13 retractions, and 10 stable transmissions were observed. In 12 paternal transmissions, the alleles expanded in 4, retracted in 4, and 4 were stable. In 21 maternal transmissions, there were 9 retractions and 6 expansions. No statistical difference in retraction frequency was found between maternal and paternal transmission (p=0.69), but our data indicate that retraction may occur more often during paternal transmissions than during maternal transmission. The alleles from the diseased father showed retraction in one case, and were stable in the other.
