# Supplementary material for: Friedreich ataxia in Norway – an epidemiological, molecular and clinical study
Source: Orphanet J Rare Dis. 2015 Sep 4;10:108. doi: 10.1186/s13023-015-0328-4 (PMC4559212; doi:10.1186/s13023-015-0328-4)
Supplement: Additional file 2: — ROC curves based on measuring frataxin from whole blood in FRDA patients, carriers and healthy controls. (DOCX 38 kb) [file 13023_2015_328_MOESM2_ESM.docx]

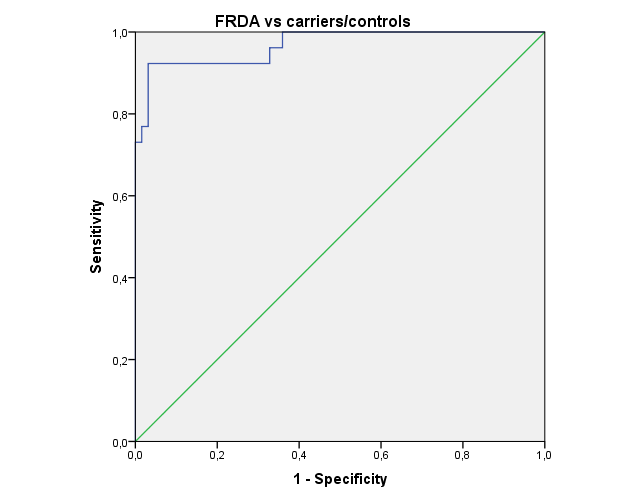


Area under ROC curve = 0.968


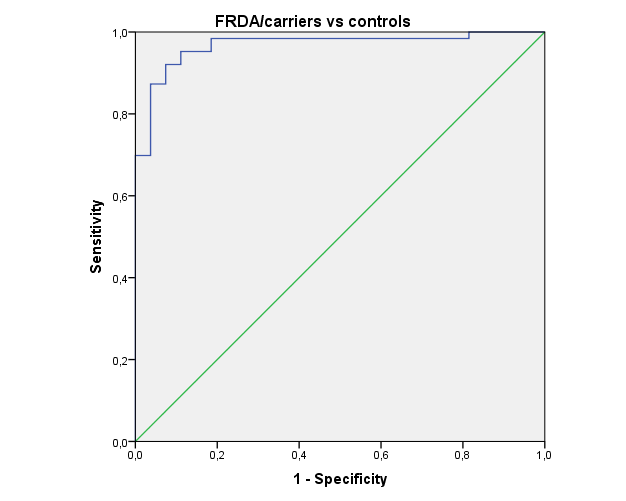


Area under ROC curve = 0.968

**Additional File 2:** ROC curves based on measuring frataxin from whole blood in FRDA patients, carriers and healthy controls
